# Wideband Spectrum Sensing: A Bayesian Compressive Sensing Approach

**DOI:** 10.3390/s18061839

**Published:** 2018-06-05

**Authors:** Youness Arjoune, Naima Kaabouch

**Affiliations:** Electrical Engineering Department, University of North Dakota, Grand Forks, ND 58202, USA; naima.kaabouch@engr.und.edu

**Keywords:** cognitive radio, compressive sensing, wideband spectrum sensing, software defined radio, Bayesian compressive sensing, autocorrelation, probability of detection, probability of false alarm

## Abstract

Sensing the wideband spectrum is an important process for next-generation wireless communication systems. Spectrum sensing primarily aims at detecting unused spectrum holes over wide frequency bands so that secondary users can use them to meet their requirements in terms of quality-of-service. However, this sensing process requires a great deal of time, which is not acceptable for timely communications. In addition, the sensing measurements are often affected by uncertainty. In this paper, we propose an approach based on Bayesian compressive sensing to speed up the process of sensing and to handle uncertainty. This approach takes only a few measurements using a Toeplitz matrix, recovers the wideband signal from a few measurements using Bayesian compressive sensing via fast Laplace prior, and detects either the presence or absence of the primary user using an autocorrelation-based detection method. The proposed approach was implemented using GNU Radio software and Universal Software Radio Peripheral units and was tested on real-world signals. The results show that the proposed approach speeds up the sensing process by minimizing the number of samples while achieving the same performance as Nyquist-based sensing techniques regarding both the probabilities of detection and false alarm.

## 1. Introduction

The growing number of wireless devices, the increasing demand for higher data rates, and the current static allocation of the radio spectrum are the leading causes of the radio spectrum scarcity problem. To address this issue and to enhance access to the radio spectrum, cognitive radio technology has been proposed as a viable solution. A cognitive radio system is a smart wireless communication system that can sense the radio spectrum, detect unused spectrum holes, and adjust dynamically its transmission parameters to access free frequency channels without causing any harmful interference to the licensed users or primary users [1,2,3]. In this regard, spectrum sensing plays a crucial role in the 3-process cognitive cycle by making cognitive radio systems aware of their radio environments. Over the last decade, several sensing techniques have been proposed to detect the primary user activities. Examples of these techniques include energy detection [4], cyclostationary detection [5], and matched filter detection [6]. Energy detection is a simple technique as it does not require prior knowledge about the primary user signal. However, it is sensitive to noise as it does not distinguish between samples coming from the signal and those coming from noise. Cyclostationary-based detection is robust to noise and has high detection performance compared with energy detection. Matched filter-based detection requires some prior knowledge about the primary user signal as it compares the received samples to some saved pilots from this signal. This technique is not practical as this information about the signal is often unavailable. 

These techniques aim at detecting spectral opportunities over narrow frequency bands; however, cognitive radio systems aim to exploit spectral opportunities over broad frequency bands ranging from hundreds of megahertz to hundreds of gigahertz and consequently these sensing techniques cannot be applied directly to perform wideband spectrum sensing.

Wideband spectrum sensing is one of the challenges facing researchers to design next-generation communication systems because this process requires a high sampling rate, which results in a high processing time and energy consumption. In conventional communication systems, this sensing is performed by an Analog-to-Digital Converter (ADC) working at the Nyquist rate leading to a high sampling rate and implementation complexity [7]. For instance, to sense a wideband signal over broad frequency bands ranging from 0 to  6 GHz, this signal has to be sampled with a high-resolution Analog-to-Digital Converter working at the Nyquist rate, which needs to be at least twice the maximum frequency of the band-limited signal, meaning 12 GHz or higher. The implementation of Nyquist-based sensing techniques is thus impractical for wideband spectrum sensing because of the hardware limitations and the computational cost. 

Several sensing approaches have been proposed to perform wideband spectrum sensing [8,9,10,11]. These approaches perform wideband spectrum sensing using the Nyquist rate. For instance, the authors of [8,9,10,11] performed spectrum measurements to find the spectrum usage pattern for broad frequency bands. To overcome the limitations of the models proposed in [8,9,10,11], some recent innovative techniques have been proposed such as wavelet-based detection [12,13,14], multi-band joint detection [15], and filter-band-based sensing [16,17,18]. The wavelet-based detection approach is an edge-based detection that characterizes the edges of the occupied channels. Using this information wavelet-based detection techniques divide the wideband spectrum into several elementary building blocks, and then a wavelet transform is applied to detect irregularities in the structure of the spectrum, which carry valuable information about the occupied channels and their frequency locations. These techniques introduce a significant latency. One way to reduce this latency is to jointly sense several bands. In multi-band joint detection, a secondary user simultaneously senses several sub-bands, and for each band, the energy of the received samples is calculated. Then, it sets optimal thresholds for all the sub-bands to maximize the likelihood of detection and minimize the likelihood of false alarm. Solving the optimization problem to find the optimal thresholds introduces a large latency instead of reducing it. In filter bank-based detection, several band-pass filters are used. At the output of each filter, an energy detector is used to detect the activity of the primary user. All the techniques mentioned above have limitations such as a high sampling rate, large latency, and high power consumption. In addition, some of these techniques have a high hardware cost. Moreover, in most of these research studies, energy detection was used to sense the frequency channels and to estimate their occupancies. However, energy detection is highly sensitive to noise, as it does not distinguish between the noise and signals; thus, this technique is inefficient particularly at low SNR values [19].

Uncertainty is yet another problem that affects wideband spectrum sensing. The authors of [20] proposed a Bayesian model to estimate the occupancy of frequency channels over broad bands and handle uncertainty. The model was implemented using GNU radio software and USRP units and was tested in real-world scenarios. Energy detection, autocorrelation, and correlation-based Euclidian Distance techniques were used for sensing these frequency channels ranging from 825 MHz to 5.8 GHz. Their results show that scanning the wideband spectrum requires a high processing time, which is not acceptable for the current or next-generation communication systems. Recently, the authors of [21] proposed an improved probabilistic model based on a Bayesian network to handle uncertainty and increase the probability of detection of these sensing techniques by estimating the level of noise. Similar to the previous studies, this method samples all the channels at the Nyquist rate, which results in a high processing time and computation cost. The authors of [22] proposed a Bayesian compressive sensing with a circulant matrix for narrow-band spectrum sensing to handle uncertainty. The proposed approach was not used for wideband spectrum sensing and it has been evaluated using only the mean square error, the recovery error, and processing time. The application of this Bayesian compressive sensing to perform spectrum sensing was not evaluated using probabilities of detection and false alarm.

To speed up the process of sensing the wideband spectrum, compressive sensing has been proposed [22,23,24]. This paradigm reduces the number of measurements to be sampled and recovers the original sparse signal from these few measurements [25]. It involves three main processes: sparse representation, measurement or encoding, and sparse recovery or decoding. Compressive sensing theory relies on two fundamental concepts: sparsity and mutual coherence [26]. These two conditions are necessary to ensure that the sparse recovery problem, which is an undetermined linear system, can be solved. In wireless communication, most of the signals are sparse in the frequency domain, as a handful of frequency channels are continuously occupied by their primary users while others are not or are sparsely used. This characteristic has enabled the application of compressive sensing for wideband spectrum sensing [22,23,24]. 

Tian et al. [27] introduced compressive sensing theory for sensing the wideband spectrum. Since then, several works have been proposed in this context [22,24,27,28,29,30,31,32,33,34,35]. For instance, the authors of [3] suggested an Analog-to-Information converter (AIC) that consists of a pseudo-random number generator, which produces a few measurements that are used to recover the sparse signal. This approach reduces the high sampling rate; however, it presents some drawbacks such as the high computational complexity because of the large size of the measurement matrix, and the performance of the AIC model can be easily affected by design imperfections. Also, the authors did not investigate how the number of measurements can be selected. To address this issue, the authors of [33] introduced a two-step compressive sensing-based model for reducing the sampling rate. The first step of this scheme consists of estimating the sparsity level of the wideband signal and accordingly adjusting the number of measurements used for sampling in the second step. This model introduced extra complexity to the wideband spectrum sensing by performing the estimation of the sparsity level. The authors of [34] proposed an approach in which they adjusted the number of measurements interactively until reaching a desired signal recovery error, which introduced further complexity to the model. To reduce the complexity of these previous approaches, the authors of [35] presented a framework that combines compressive spectrum sensing with geo-location database. This database provides the system with the necessary information about the occupancy of a band of interest to estimate its sparsity level and, accordingly, find the accurate number of measurements to guarantee the best recovery of the wideband signal. This model is impractical since the database has to be updated in real-time to provide the system with the right information.

Most of the proposed compressive-sensing techniques have been evaluated in either a noise-free environment or using simulations in non-realistic scenarios. For instance, the authors [26] evaluated their compressive sensing model using probabilities of detection and false alarm using simulation and under high values of signal to noise ratio, SNR = 15 dB. The performance of the algorithm under a low signal-to-noise ratio was not considered. It would be more reasonable to test the behavior of the models under low signal-to-noise ratios and on real-world signals, especially if these models are based on methods sensitive to noise such as energy detection. In addition, the validation of the proposed model using only simulations with Matlab is not sufficient. Indeed, several parameters that are assumed to be constant in simulations are random in real-world scenarios. 

A few research works have been conducted on the implementation of compressive sensing to investigate the reliability of this new sensing framework. For instance, the authors of [36,37] proposed a modulated wideband converter model for multi-channel CS-based sensing. The authors of [33] proposed an analog-to-information sampler to implement compressive sensing. The authors of [27] tested their wideband spectrum sensing technique on real-time TV white space signals obtained by a CRFS RFeye node to validate the proposed model; however, this model was not tested and the evaluation of the proposed model in an uncontrolled environment is very difficult. Thus, there is a strong need for validating the compressed wideband sensing techniques using real-world experiments and to test these techniques on real signals to ensure the reliability of these sensing techniques. 

In this paper, we propose a Bayesian compressive approach for sensing the wideband radio spectrum. The proposed scheme is based on Bayesian compressive sensing via fast Laplace prior (BCS-LP) to recover the wideband signal from a few measurements combined with an autocorrelation-based detection technique to obtain an accurate sensing decision. Unlike most of the existing techniques, the proposed model uses an autocorrelation-based detection technique because it is insensitive to noise. The proposed approach was implemented using GNU Radio software and universal software radio peripheral (USRP) units and was extensively tested on real-time signals. The experimental setup uses real-time signal transmitted and recorded using the USRP and GNU radio. The level of the signal-to-noise ratio was measured using a method based on the eigenvalues of the sample covariance matrix of the received signal [24,25,26,27]. 

The contributions of this paper can be summarized as follows:

- A review of the wideband sensing techniques
- The mathematical model of the Bayesian compressive sensing-based method
- An experimental setup using software defined radio units
- An evaluation of the proposed approach using several metrics
- A Comparison between the proposed approach and three Nyquist-based sensing techniques

The rest of this paper is structured as follows. Section 2 describes the proposed model and its mathematical model as well as the experimental setup. Section 3 presents and discusses the obtained results. Conclusions and perspectives are drawn in Section 4.

## 2. Methodology

The proposed approach for sensing the wideband spectrum is based on Bayesian compressive sensing and an autocorrelation-based detection technique. During this sensing, the receiver only captures a few samples. Then, the Bayesian-based recovery technique recovers the wideband signal from these few measurements followed by the autocorrelation-based detection technique to obtain the sensing decision. In the first part of this section, the mathematical model of the proposed approach is described, and in the second part, the implementation of this approach is described as well as the experimental setup used to test the model.

### 2.1. Mathematical Model

The wideband signal considered is divided into *N* sub-bands. Then, the received signal at the secondary user can be expressed as:

(1) $$y\left(t\right)=\sum_{n=1}^{N}x_{n}\left(t\right)*h_{n}\left(t\right)+w\left(t\right),$$

where $x_{n}\left(t\right)$ denotes the signal of the $n^{th}$ primary user, $h_{n}\left(t\right)$ denotes the channel response, and w(t) denotes an additive white Gaussian noise.

In the frequency domain, this signal can be expressed as:

(2) $$Y\left(f\right)=\sum_{n=1}^{N}D_{h}*x_{n}\left(f\right)+w\left(f\right),$$

where $D_{h}\;$ denotes a diagonal N×N channel gain matrix.

At a time  t, only a few M channels are occupied where  M<<N. Consider S the set of occupied channels. Then, for all channels that do not belong to the set  S, their corresponding signals are zero. Thus, the received signal can be reduced to:

(3) $$y\left(t\right)=\sum_{n\in S}x_{n}\left(t\right)*h\left(t\right)+w\left(t\right),$$

(4) $$Y\left(f\right)=\sum_{n\in S}D_{h}*x_{n}\left(f\right)+w\left(f\right),$$

where Y(f) is sparse in the frequency domain, which makes the application of compressive sensing work.

The received signal is then reduced using a sensing matrix to obtain the vector of measurements. A sensing matrix, which has to satisfy the Restricted Isometry Property, is used to select the few measurements from the sparse signal. The vector of measurements is then given by:

(5) $$z_{f}=\Phi y_{f},$$

where x denotes an N×1 vector, $z_{f}$ denotes the M×1 measurements vector, Φ denotes an M×N sensing matrix, and $y_{f}$ is the projection of the received signal on the frequency domain.

The signal is then reconstructed from the set of measurements $z_{f}$ using the Bayesian via Fast Laplace prior technique, which uses prior knowledge of the sparse signal in order to recover the original sparse signal. 

The recovery formulation is given as

(6) $$\underset{y_{f}}{\min}||y_{f}{||}_{1}\;s.t.\;z_{f}=\Phi y_{f},$$

The signal is then reconstructed from the set of measurements $z_{f}$ using the Bayesian via Fast Laplace prior technique, which uses a Bayesian inference on the sparse signal in order to recover the original sparse signal.

Bayesian compressive sensing requires determining a joint probability distribution function (pdf) of the hierarchical model $f\left(y_{f},\gamma ,\beta ,z_{f}\right)$. This pdf function is defined as:

(7) $$f\left(y_{f},\gamma ,\beta ,z_{f}\right)=f\left(z_{f}/y_{f},\beta \right)\;\cdot f\left(y_{f}/\gamma \right)\cdot \;p\left(\gamma \right)\cdot \;p\left(\beta \right),$$

where $\beta =\frac{\sigma^{2}}{2}$ denotes the inverse of noise variance, γ and β denote hyper-parameters, and the vector of observations $z_{f}$ is assumed to underline a normal probability distribution with zero mean and variance equal to $\beta^{-1}$. Thus, the conditional probability of measurement vector $z_{f}$ given the sparse signal $y_{f}$ can be expressed as:

(8) $$f\left(z_{f}/y_{f},\beta \right)=N\left(z_{f}/\phi y_{f},\beta^{-1}\right),$$

The hyper-parameter β underlines a gamma probability distribution function, and this pdf can be given by:

(9) $$f\left(\beta /a^{\beta },b^{\beta }\right)=\Gamma \left(\beta /a^{\beta },b^{\beta }\right),$$

The signal model is equivalent to using a Laplace prior on the coefficients $y_{f}$; thereby, we can formulate the probability density function as:

(10) f(yf|γ)=(γ2)Nexp(−γ2||yf||1),

The Bayesian inference is given by:

(11) $$f\left(x,\gamma ,\beta ,\lambda /z_{f}\right)=f\left(y_{f}/z_{f},\gamma ,\beta ,\lambda \right)f\left(\gamma ,\beta ,\lambda /z_{f}\right),$$

Since $f\left(y_{f}/z_{f},\gamma ,\beta ,\lambda \right)\propto f\left(y_{f},z_{f},\gamma ,\beta ,\lambda \right)$, then the distribution $f\left(y_{f}/z_{f},\gamma ,\beta ,\lambda \right)$ is a multivariate Gaussian distribution $N\left(y_{f}/\mu ,\sum^{\text{​}}\right)$ with the parameters:

(12) $$\mu =\sum^{\text{​}}\beta \phi^{T}y,\sum^{\text{​}}={\left[\beta \phi^{T}\phi +\wedge \right]}^{-1},\mathrm{and}\;\wedge \;=diag\left(1/\gamma_{i}\right),$$

$f\left(\gamma ,\beta ,\lambda /z_{f}\right)=[f\left(\gamma ,\beta ,\lambda ,z_{f}\right)/f\left(z_{f}\right)]\;\propto \;f\left(\gamma ,\beta ,\lambda ,z_{f}\right)$ is used to estimate the hyperparameters. We estimate these hyperparameters by maximizing the joint distribution $f\left(\gamma ,\beta ,\lambda ,z_{f}\right)\;$ or its logarithm  l:

(13) $$\begin{array}{l} l=\log\left(f\left(\gamma ,\beta ,\lambda ,y\right)\right)=-\frac{1}{2}log\left|C\right|-\frac{1}{2}y^{T}C^{-1}y+Nlog\left(\lambda \right)-\frac{1}{2}\sum^{\text{​}}\gamma_{i}+\frac{\vartheta }{2}\log\left(\frac{\vartheta }{2}\right)-\log\left(\left(\frac{\vartheta }{2}\right)\right)\\ +\left(\frac{\vartheta }{2}-1\right)\log\left(\lambda \right)-\frac{\vartheta }{2}\lambda +\left(a^{\beta }-1\right)\log\left(\beta \right)-b^{\beta }\beta , \end{array}$$

The updates of other parameters can be determined by setting the two partial derivatives of the logarithm of the joint probability density function $f\left(\gamma ,\beta ,\lambda ,z_{f}\right)$ to zero. This can be expressed as:

(14) $$\frac{dl}{d\lambda }=0\mathrm{and}\frac{dl}{d\beta }=0,$$

Thus, we find expressions for these two hyper-parameters, which are given by:

(15) $$\beta =\frac{\frac{N}{2}+a^{\beta }}{\frac{\langle ||y-\phi x^{2}||\rangle }{2}+b^{\beta }},$$

$$\lambda =\frac{N-1+\frac{\vartheta }{2}}{\sum_{i}\frac{\gamma_{i}}{2}+\frac{\vartheta }{2}},$$

Once we determine the hyper-parameters, we can find an expression for the joint probability density function $f\left(y_{f},\gamma ,\beta ,z_{f}\right)$, and consequently we can solve the recovery problem given by Equation (6).

Once the wideband signal is recovered, a detection technique has to be applied to obtain the sensing decision. In our model, we performed the detection using autocorrelation-based sensing detection because of its advantages compared to other techniques [4]. This sensing technique uses some cyclostationary features of the signal samples to distinguish the transmitted signal from noise [5]. The autocorrelation of the received signal is thus analyzed to decide between two states: the signal is considered absent, denoted by $\;{ℋ}_{0}$, and the signal is considered present, denoted by $\;{ℋ}_{1}$.

(16) $$\;{ℋ}_{0}:\;y\left(n\right)=w\left(n\right),$$

(17) $$\;{ℋ}_{1}:\;y\left(n\right)=s\left(n\right)+w\left(n\right),$$

where y(n) denotes the received signal, *s*(*n*) denotes the primary user signal, and w(n) is an additive white Gaussian noise.

The statistical covariance matrices of the vector y(n) and s(n), which are denoted by $R_{y}$ and $\;R_{s}$, respectively, are given by:

(18) $$R_{y}=E\left[y\left(n\right)y^{T}\left(n\right)\right],$$

(19) $$R_{s}=E\left[s\left(n\right)s^{T}\left(n\right)\right],$$

where *E* denotes the expectation and the ${\left(\cdot \right)}^{T}$ represents the transpose operator.

The matrix $R_{y}$ can be expressed as follows:

(20) $$R_{y}=R_{s}+\sigma_{\eta }{}^{2}Ι,$$

where $\sigma_{\eta }{}^{2}$ denotes the variance of the noise and *I* represents the identity matrix.

If the signal s(n) is absent, the off-diagonal entries of $R_{x}$ are zeros ${\left(R_{y}\right)}_{ii}=0$ as well as $\;R_{s}=0$. The authors of [5] proposed a ratio between the sum of all elements of $\;R_{y}$, $\;T_{1}$, and the sum of its diagonal elements, $T_{2},$ as a metric to detect the presence or the absence of the signal. If the ratio is equal to 1, it means that there is no signal, and if the ratio is greater than 1, then the signal is considered present. $T_{1}$ and $T_{2}$ are defined as:

(21) $$T_{1}=\frac{1}{L}\sum_{j}^{L}\sum_{i}^{L}\left|r_{ij}\right|,$$

(22) $$T_{2}=\frac{1}{L}\overset{L}{\underset{j}{\sum }}\left|r_{ij}\right|$$

where $r_{ij}$ denotes the *ij*-th element of the matrix $R_{y}$.

### 2.2. Experimental Setup

The proposed approach and the Nyquist-based model were implemented using GNU Radio and USRP units. GNU Radio is open source software that contains several digital signal-processing blocks, which can be used to implement radio subsystems. To create a new customized block in GNU Radio, either C++ or Python language can be used. USRP units are Software Defined Radio devices designed for RF applications and can be interfaced with GNU Radio software, which has a USRP Hardware Driver (UHD) block allowing the computer to capture the samples coming from these units. 

The USRP units used in this work have WBX daughterboards with a maximum effective bandwidth of 25 MHz. Given a wideband signal of bandwidth higher than 25 MHz, USRP units cannot sense the whole bandwidth spectrum in one-step. The procedure considered is a multistep process in the frequency domain. This procedure defines the spectrum range to be sensed, and then the algorithm changes the central frequency of the receiver to obtain the samples to be processed. This process is repeated for all frequencies within the testing range.

**Figure 1:**
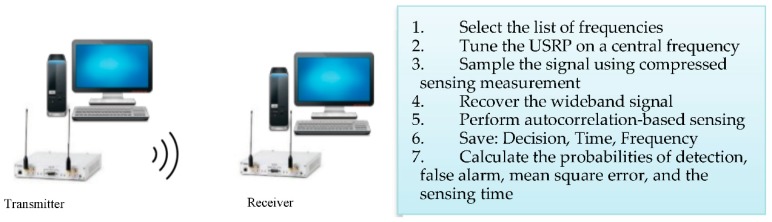
Experimental setup using software defined radio units and GNU Radio.

This experimental setup is illustrated in Figure 1. Two sets of USRP units and two computers have been used. In the transmitter, consisting of one USRP and one computer, we emulate the signal generated by the primary user, in which the characteristics of the signal are adjusted for transmission. Random Additive White Gaussian noise (AWGN) is added to the signals before transmission. 

The second USRP, which acts as the receiver, is tuned to a center frequency. Then, the samples corresponding to this center frequency are captured by the UHD block and saved in a Python Numpy Array using the block printer_f. For each center frequency, the measurement matrix is applied to the array that contains the samples captured from the USRP unit to obtain only a few samples. The sensing matrix used is a random Gaussian matrix. Based on these few samples and a Toeplitz measurement matrix, the signal is reconstructed via the BCS-LP recovery technique, and then the autocorrelation technique is applied to the recovered signal to determine the presence or the absence of the primary user. All collected measurements are saved in a Python Numpy array. For comparison purposes, standard sensing is also performed for each center frequency, which skips the compressive sensing part and applies the autocorrelation directly to the received signal. The same operation is done for all center frequencies listed in Table 1.

Table 1 shows the list of frequency channels of four bands considered for this sensing. Their ranges, the spacing between the frequency channels of the same band, and the number of channels in each band are presented. These frequencies were chosen for their high use in real-world communication scenarios.

## 3. Results

Several experiments were performed to evaluate the efficiency of the proposed model. In the following, we present the results of the comparison between our proposed approach, Bayesian compressed sensing model, and the Nyquist-based sensing technique. The choice of the Bayesian recovery algorithm is justified by the results of a comparison between several recovery techniques [38] conducted in our previous work. These algorithms are basis pursuit (BP), gradient descent (Grades), orthogonal matching pursuit (OMP), iterative hard thresholding (IHT), and Bayesian via relevance vector machine (BCS-RVM). These algorithms were chosen because they best represent techniques from three categories: convex and relaxation, greedy, and Bayesian category. Metrics such as the recovery error and recovery time were used for the performance comparison. As summarized in Table 2, this work showed that OMP is the fastest algorithm, but it has a high recovery error; BP shows small recovery error, but it requires more recovery time; and Bayesian-based techniques are the balance between low recovery error and short recovery time. 

The performance of compressive sensing techniques is affected by the measurement matrix used. In a previous work that compared the performances of measurement matrices [39], we show that the Toeplitz matrix outperforms the other measurement matrices. It has the advantages of reducing the randomness because of its deterministic construction and it satisfies the restricted isometry property, which can result in more accurate recovery and faster sensing process. For this reason, we used the Toeplitz matrix with the Bayesian recovery to perform compressive sensing.

In this work, to evaluate the wideband spectrum sensing models, several evaluation metrics are used, which are the probability of detection ($P_{d}$), the probability of false alarm ($P_{fa}$), the mean square error  (MSE), and the sensing time.

The probabilities of detection and false alarm are given by:

(23) $$P_{d}=\frac{N_{D}}{N_{T}},$$

(24) $$P_{fa}=\frac{N_{FA}}{N_{T}},$$

where $N_{D}$ is the number of total detections, $N_{FA}$ is the number of total false alarms, and $N_{T}$ is the total number of trials.

The mean square error is given by:

(25) $$\mathrm{MSE}=\frac{1}{N}\sum^{\text{​}}{\left(Y-\hat{Y}\right)}^{2},$$

where *Y* is the received signal using Nyquist-based sensing and $\hat{Y}$ is the recovered signal.

Table 3 summarizes the implementation parameters. The PU signal is a BPSK modulated signal with a transmission power of P=−70 dB, and the values of SNR is varied from  −20 dB to 20 dB. The absence of the PU signal is emulated by turning the transmitter off. In order to calculate the probabilities of detection and false alarm, we have considered a number of trials equal to  100. 

During the experiment, we encountered several challenges. The first challenge was how to generate signals with different signal-to-noise-ratio values because the noise is time-varying and cannot be controlled. We solved this problem by connecting both the USRPs of the receiver and the transmitter directly using a radio frequency (RF) cable, and we change the level of noise at the transmitter side. Another challenge was how to make sure that the SNR we are targeting is the one that we have created. To overcome this challenge, we used a noise measurement method based on the eigenvalues of covariance matrix of the received samples [40,41]. This technique allows one to estimate the noise power and the signal power in a signal at the receiver side.

**Figure 2:**
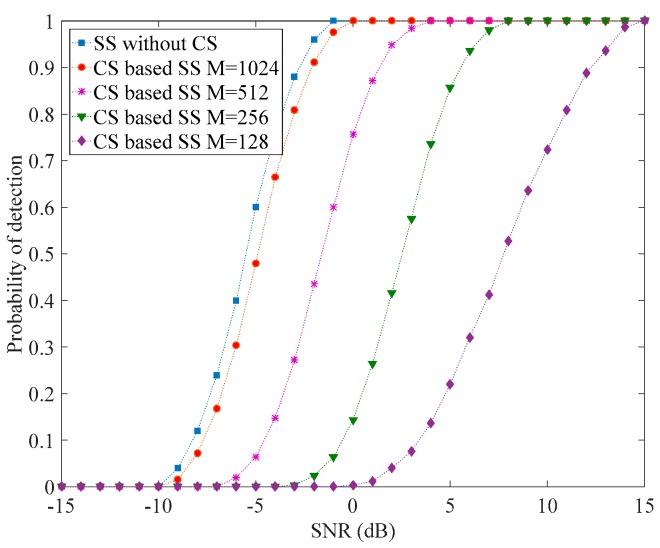
Probability of detection versus SNR for two approaches, normal sensing and CS based sensing, of the channel 2.437 GHz for a probability of false alarm *P_fa_* = 0.1, number of samples *N* = 2048, and four values of the number of measurements, *M* = 128, 256, 512, and 1024.

**Figure 3:**
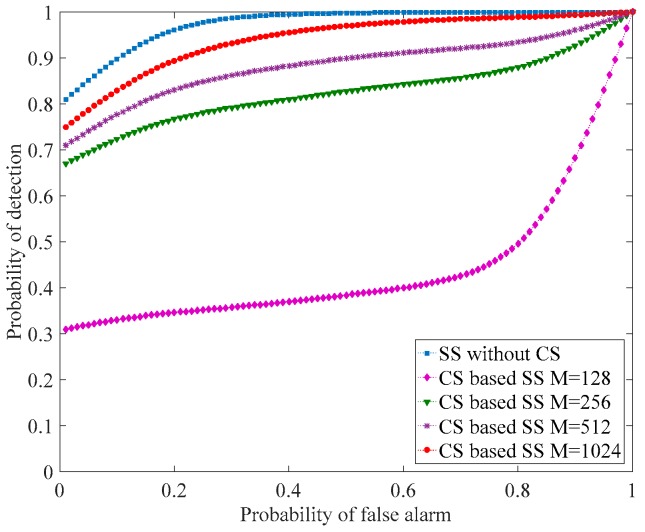
Probability of detection versus the probability of false alarm for the two approaches, normal sensing and CS based sensing, of the channel 2.437 GHz. In this experiment, SNR = −5 dB, number of samples *N* = 2048, and number of measurements, *M* = 128, 256, 512, and 1024.

**Figure 4:**
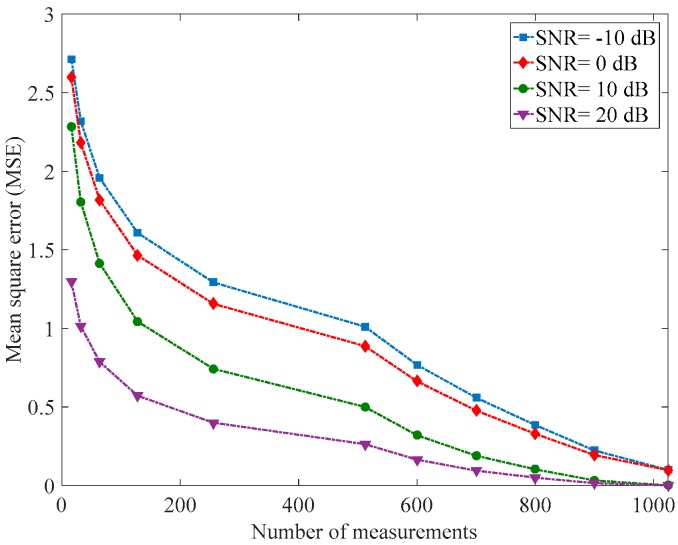
Mean square error of the proposed approach versus the number of measurements for the SNR values −10, 0, 10, and 20 dB.

**Figure 5:**
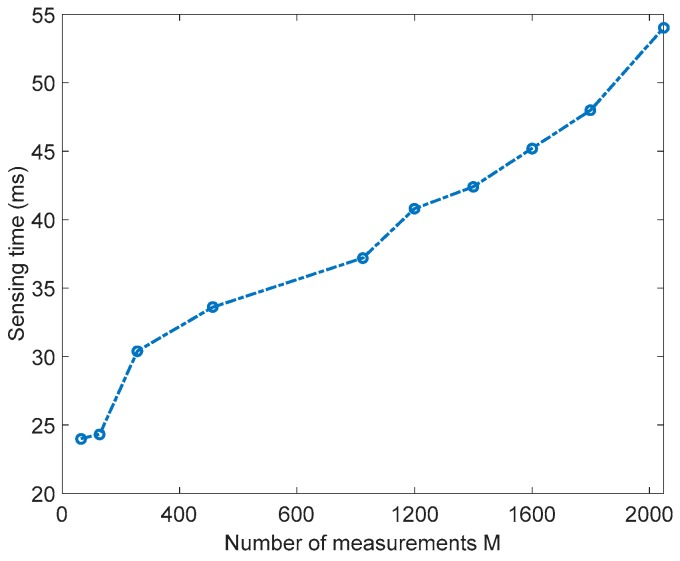
Sensing time for 20 channels of the band 2.437 GHz versus the number of measurements.

Examples of results are shown in Figure 2, Figure 3, Figure 4 and Figure 5. Figure 2 presents a comparison between the probability of detection versus SNR for CS based sensing using different numbers of measurements and the probability of detection versus SNR of the Nyquist-based sensing technique. The SNR values range from −20 dB to  +20 dB. This figure also shows that the probability of detection increases as SNR increases for both approaches, the proposed approach and Nyquist-based sensing, to reach 100% for  SNR=0 dB. It can also be observed that the probability of detection with CS based sensing reaches 100% for lower SNR values as the number of measurements increases. For a number of measurements  M=128, the probability of detection reaches 100% for SNR=15 dB and for  M=1024, the probability of detection reaches 100% for  SNR=0 dB. If we reduce the number of measurements by  50%, the probability of detection decreases by 10% for SNR=−5dB and by 5% for SNR=−2 dB, but remains constant for SNR higher than 0 dB. This means that if we are able to estimate the value of  SNR, we can set the number of measurements to a specific value and still obtain the same value of probability of detection. For instance, for  SNR=3 dB, the number of measurements can be reduced to M=512 and the detection performance remains the same as the one of the Nyquist-based sensing.

Figure 3 shows the probability of detection versus the probability of false alarm for standard sensing and compressive sensing based techniques using different numbers of measurements. One can see that the probability of detection increases as the probability of false alarm increases to reach 100% for $P_{fa}=30\%$ for normal sensing and $P_{fa}=50\%$ for compressive sensing based sensing for a compression ratio  M/N=50. The same figure shows that the probability of detection increases as the number of measurements increases. The lowest value of probability of detection corresponds to the number of measurements M=128 and the highest one corresponds to the number of measurements  M=1024. 

Figure 4 shows the MSE  of the proposed approach versus the number of measurements for different values of SNR. As expected, the mean square error decreases as the number of measurements increases for all values of SNR. The MSE corresponding to low SNR values is higher and the ones corresponding to high SNR values are lower. It can also be seen that the MSE increases as the SNR decreases. Hence, if we are able to estimate the value of SNR, we can choose the number of measurements accurately and then improve the detection performance of the compressive spectrum sensing technique.

Figure 5 shows the sensing time needed to scan 20 channels of the band 2.4 GHz, versus the number of measurements. As expected, the sensing time decreases as the number of measurements decreases. For instance, for the number of measurements *M* = 2048, the sensing time is 54 ms and for *M* = 1024, the sensing time is 37 ms, which means that if we decrease the number of measurements by 50%, the sensing time is also decreased by almost 32%.

Table 4 shows examples of performance comparisons of the two sensing techniques. Column a gives examples of results corresponding to the normal sensing technique. Columns  b,  c, d, and e give examples of results corresponding to the compressive sensing based sensing technique for different measurements numbers, 1024, 512, 256, and  128. As one can see, for a 10% of probability of false alarm, normal sensing (a) achieves a probability of detection of 100% for  SNR=−1 dB. However, the compressive sensing-based sensing technique achieves a detection probability of  100%, 75%, 15%, and 0% for SNR=0 dB  for the measurement numbers 1024, 512, 256, and 128, respectively. The sensing time corresponding to the normal sensing based sensing technique is 54 ms while the time for the compressive sensing-based sensing technique is 37 ms, 33.6 ms, 30.4 ms, and 24.3 ms, for the measurement numbers, 1024, 512, 256, and 128, respectively. For the mean square error, the compressive sensing based sensing technique has MSE values of 0.09, 0.88, 1.156, and 1.456, for the measurement numbers, 1024, 512, 256, and 128, respectively. As a summary, one can conclude that by reducing the number of samples by 50%, the probability of detection decreases by only a maximum of 10%, but the sensing time is decreased by approximately 32%. 

Table 5 provides a comparison between several techniques. This table highlights the advantages and disadvantages of each technique. Wavelet-based detection has the advantages of reducing the sensing time compared with sequential sensing since it uses the irregularities in the edge to find the occupied channels as well as their locations. However, the sensing time of this detection approach is high because of the high sampling rate, which results in a high power consumption and complexity. This technique has also the disadvantage of low detection at low values of SNR. Multiband joint-based detection has the advantages of sensing multiple bands simultaneously, finding the optimal threshold for energy detector, and reducing the latency compared to wavelet-based detection. However, the optimization process requires an iterative process to find the optimal thresholds that increase the probability of detection while reducing the probability of false alarm. In addition, this technique uses the Nyquist rate, and consequently suffers from high complexity, large latency, and high complexity implementation. Filter bank-based detection senses the wideband spectrum using several band-pass filters, which can speed up the process of sensing compared to wavelet and multi-band based detection techniques. However, using energy detectors at the output of each filter does not give accurate sensing results as energy detection is very sensitive to noise. Moreover, filter bank-based detection has a high hardware cost since it requires several RF receiver and ADC converters, which is unpractical. Compressive sensing-based wideband spectrum sensing reduces the high sampling rate, and thus has a short processing time that can be up to 50% less than for Nyquist-based techniques while achieving the same detection performance. In addition, using autocorrelation with compressive sensing has the advantage of coping with noise uncertainty. 

## 4. Conclusions

In this paper, we have proposed a novel approach for speeding up the process of wideband spectrum sensing, handling uncertainty, and identifying the free radio channels. This approach uses Bayesian compressive sensing combined with the autocorrelation-based detection. The proposed technique was implemented using GNU Radio and USRP units. Numerous experiments were performed to validate the proposed model using metrics, including the probability of detection, probability of false alarm, mean square error, and sensing time. The efficiency of the proposed model was compared with that of several Nyquist-based sensing techniques. These results show that this model speeds up the sensing process while achieving approximately the same probabilities of detection and false alarm as Nyquist-based sensing techniques. For future work, we will focus on further improving the detection performance of the compressive sensing techniques by measuring SNR and automatically selecting the number of measurements to reduce the recovery error. We plan also to investigate the application of one-bit compressive sensing for wideband spectrum sensing because of its advantages such as low complexity, fast sampling, and low storage cost as well as the robustness to noise.

## Figures and Tables

**Table 1:** List of frequencies scanned in this work.

	Start Frequency MHz	Stop Frequency MHz	Number of Channels	Spacing MHz
GSM 850	869	894	126	0.2
GSM 1900	1930	1990	126	0.2
Wi-Fi 2.4	2402	2501	30	5
Wi-Fi 5.8	5725	5880	30	5

**Table 2:** Performance comparison of recovery techniques.

Recovery Techniques	Recovery Error	Recovery Time	Complexity
Convex and relaxation techniques	minor	Long	complex
Greedy techniques	High	Short	Not complex
Bayesian recovery techniques	minor	Short	Not complex

**Table 3:** Implementation parameters.

Parameters	Values
Number of samples	2048
Modulation	BPSK
Transmission power	−70 dBm
FFT size	512
SNR range in dB	−20 to 20
Number of trials	100

**Table 4:** Performance Comparison of sensing techniques.

Evaluation Metrics	Nyquist Sensing (a)	Sensing with CS M/N=50% (b)	Sensing with CS M/N=25% (c)	Sensing with CS M/N=12.5% (d)	Sensing with CS M/N=6.25% (e)
Probability of detection SNR= 0 dB and $P_{fa}=10\%$	100%	100%	75%	15%	0%
Processing time in (ms)	54	37	33.6	30.6	24.33
MSE for SNR=0 dB	No recovery	0.09	0.88	1.15	1.46

**Table 5:** Comparison between wideband spectrum sensing techniques.

Wideband Sensing Technique	Advantages	Disadvantage
Wavelet-based detection [12,13,14]	Reduced latency compared to sequential sensing Find the frequency locations of the occupied channels No sensing of the sub-bands one-by-one is required	High latency High power consumption High sampling rate High complexity of implementation Low detection at low SNR values
Multi-band joint-based detection [15]	Optimize the selection of the threshold for energy detector The detection is performed jointly Reduced latency compared with wavelet-based detection	The optimization introduces large latency High sampling rate High complexity implementation
Filter bank-joint detection [16,17,18]	Moderate latency High detection performance	Hardware cost High sampling rate Low detection performance at low SNR values
Compressive sensing-based detection	Reduced latency and sampling rate compared with Nyquist-based sensing Insensitive to noise uncertainty Reduce the processing time Power consumption is reduced The same detection performance as Nyquist-based detection with lost cost	The performance of this techniques depends on the accuracy of the recovery process Estimation of the sparsity level and recovery introduce extra latency
